# SESN2 inhibits tubular exosome secretion and diabetic kidney disease progression by restoring the autophagy‒lysosome pathway

**DOI:** 10.7150/ijbs.109799

**Published:** 2025-06-20

**Authors:** Zongji Zheng, Jiaqi Chen, Xiaoquan Xue, Xiaoqin Ma, Shuting Zhang, Ming Wang, Yaoming Xue, Yijie Jia

**Affiliations:** 1 Department of Endocrinology & Metabolism, Nanfang Hospital, Southern Medical University, Guangzhou, China.; 2 Department of Endocrinology, Guangdong Provincial People's Hospital (Guangdong Academy of Medical Sciences), Southern Medical University, Guangzhou, China.; 3 School of Traditional Chinese Medicine, Southern Medical University, Guangzhou, China.

**Keywords:** Diabetic kidney disease, SESN2, exosomes, autophagy, lysosome

## Abstract

During diabetic kidney disease (DKD), tubulointerstitial fibrosis persists, although several methods have been applied to reduce albuminuria levels. In this research, we found that bovine serum albumin (BSA)-induced renal tubular cell injury could also spread to normal tubular cells through exosomes, which may explain why tubulointerstitial fibrosis persists. Our previous studies revealed that SESN2 overexpression alleviates tubular dysfunction. In this study, we showed that SESN2 overexpression in donor HK2 cells interrupted this "doom loop" and confirmed that SESN2 may mediate this process by reducing exosome secretion. By using RNA-seq and IP-MS, we found that SESN2 could inhibit BSA-induced Rab-7a ubiquitination, thus promoting autophagosome and lysosome fusion and accelerating MVB degradation. We also showed that SESN2 promotes the nuclear translocation of TFEB through the mTOR pathway, thus further alleviating lysosomal function and promoting MVB degradation. We also found that SESN2 not only slowed DKD progression but also promoted renal tubular cell secretion of protective exosomes, which also slowed DKD progression. In conclusion, SESN2 can interrupt the progression of albuminuria-induced tubular injury by inhibiting exosome secretion and promoting MVB degradation. Thus, SESN2 may be a new therapeutic target for DKD treatment.

## Introduction

Diabetic kidney disease (DKD) is the leading cause of end-stage renal disease, the incidence rate of which increased from 19.0% in 2000 to 29.7% in 2015 worldwide 1. Increased albuminuria reflects early kidney injury and progression throughout DKD 2. After reabsorption by proximal cells, a major fraction of the proteins undergo transcytosis and are delivered intact into the blood, while others are degraded to amino acids by lysosomal proteases 3. With the development of DKD, the protein content increases, and lysosomal rupture and tubular injury are induced, thus leading to tubulointerstitial fibrosis 4. Although several methods are used to reduce albuminuria, the process of DKD continues to progress 5. These phenomena indicate that although reduced, albuminuria is still effective, though the mechanisms are unknown. In fact, tubular injuries are generally scattered during the early stages and expand during disease progression, and in recent years, several studies have reported that injured tubules can “infect” normal tubules and subsequently amplify tubular damage, which may explain why DKD inevitably progresses 6-8. Thus, understanding the pathophysiological changes of renal tubular cells after exposure to albuminuria and how to interrupt this "doom loop" might lead to the development of new therapeutic measures for DKD.

Exosomes are 30-150 nm nanovesicles with lipid bilayer membranes that enclose various biomolecules derived from cells, such as RNA, DNA and proteins 9. An increasing number of studies has shown that exosomes play a key role in mediating signal transduction between renal cells 10, 11. Several previous studies have shown that albuminuria promotes tubular-derived exosome secretion, and albuminuria-exposed tubular cells can induce other renal cells to differentiate into inflammatory and fibrotic phenotypes through exosomes 12-14. Thus, inhibiting tubular cell exosome secretion may reduce injury-related signal transduction from injured tubular cells. The complex processes involved in exosome secretion; the formation of precursor multivesicular bodies (MVBs); and transport, docking, and fusion with the cell membranes all influence exosome secretion 15. However, a potential target for inhibiting tubular cell exosome secretion during DKD still needs to be fully elucidated.

Sestrin2 (SESN2), a member of the highly conserved Sestrin family of stress-inducible metabolic proteins, has been shown to be able to protect cells from various stresses 16. Our previous research revealed that SESN2 could protect renal tubular cells from albuminuria-induced ER stress 17. Recent studies have reported that SESN2 has two important functions; one is to protect cells from oxidative stress, and the other is to inhibit the activity of mammalian target of rapamycin complex 1 (mTORC1), thus increasing autophagic catabolism 18. For example, SESN2 has been shown to promote neuronal survival after spinal cord injury through the AMPK/mTOR signaling pathway, which activates autophagy during endoplasmic reticulum (ER) stress 19. In another study, arsenite-induced oxidative stress suppressed SESN2 expression, thereby inhibiting autophagy independently of the AMPK/mTOR pathway 20. Moreover, under oxidative stress conditions, SESN2 was reported to bind to SQSTM1 and ULK1 and further interact with Keap1, which is degraded through the autophagy process 21. This research indicated that under stress conditions, SESN2 could regulate autophagy through distinct molecular pathways. As several studies have reported that autophagy is involved in MVB transport 22, 23, we investigated whether SESN2 could protect renal tubular cells and simultaneously inhibit exosome secretion.

In this study, we aimed to investigate whether SESN2 inhibited crosstalk between tubular cells through exosome secretion during DKD and to clarify the possible underlying mechanisms. The results demonstrated that SESN2 not only alleviates renal tubular cell injury after exposure to albuminuria but also inhibits the transport of this injury message between tubular cells. Therefore, SESN2 may serve as a promising therapeutic target for DKD patients with albuminuria.

## Results

### Exosomes from BSA-induced tubular cells promote normal tubular epithelial cell injury

**Figure 1:**
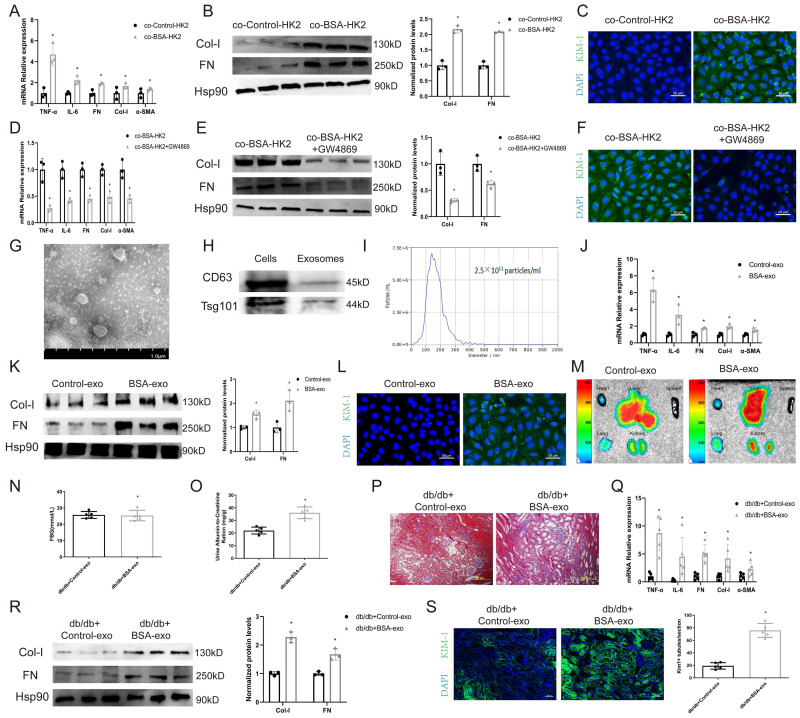
** Exosomes from BSA-induced tubular cells promote recipient tubular cell injury.** HK-2 cells were co-cultured with Control HK2 cells (co-Control-HK-2) and BSA-treated HK2 cells (co-BSA-HK2): (A) mRNA expression levels of TNF-α, IL-6, FN, Col-I and α-SMA (n=3). (B) Protein expression levels of Col-I and FN (n=3). (C) Representative immunostaining images of KIM-1. Scale bars: 50 μm. *p < 0.05 vs. the co-Control-HK-2 group. HK-2 cells were co-cultured with BSA-treated HK2 cells (co-BSA-HK2) and BSA-treated GW4869-treated HK2 cells (co-BSA-HK2+GW4869): (D) mRNA expression levels of TNF-α, IL-6, FN, Col-I and α-SMA (n=3). (E) Protein expression levels of Col-I and FN (n=3). (F) Representative immunostaining images of KIM-1. Scale bars: 50 μm. *p < 0.05 vs. the co-BSA-HK-2 group. (G) Representative TEM images of exosomes isolated from HK-2 cells. Scale bars: 1.0 μm. (H) Detection of exosome markers in both cell lysates and exosomes. (I) NTA analysis diameters of exosomes isolated from HK-2 cells. HK-2 cells were co-cultured with exosomes from Control HK2 cells (Control-exo) and BSA-treated HK2 cells (BSA-exo): (J) mRNA expression levels of TNF-α, IL-6, FN, Col-I and α-SMA (n=3). (K) Protein expression levels of Col-I and FN (n=3). (L) Representative immunostaining images of KIM-1. Scale bars: 50 μm. *p < 0.05 vs. the Control-exo group. Db/db mice were injected with Control-exo (db/db+Control-exo) and BSA-exo (db/db+BSA-exo): (M) IVIS images of different organs harvested 24 h after db/db mice were injected with Did-labelled exosomes. (N) Fasting blood glucose. (O) Urinary ACR levels. (P) Masson staining of kidney cortex. Scale bars: 100 μm. (Q) mRNA expression levels of TNF-α, IL-6, FN, Col-I and α-SMA (n=5). (R) Protein expression levels of Col-I and FN (n=3). (S) Representative immunostaining images of KIM-1(n=5). Scale bars: 50 μm. *p < 0.05 vs. the db/db+Control-exo group.

To test whether albuminuria-injured tubular cells could promote injury to normal congener cells, we cocultured BSA-treated HK2 cells (BSA-HK2) with normal HK2 cells. As shown in Figure 1A-B, coculture with BSA-HK2 cells promoted the expression of fibrosis and inflammation markers in normal HK2 cells. Moreover, immunofluorescence staining revealed that expression of kidney injury molecule (KIM)-1, a marker of injured proximal tubules, was upregulated in normal HK2 cells (Figure 1C), which indicated that BSA-HK2 cells could influence normal HK2 cells through cell-to-cell communication. We and others have reported that injured tubular epithelial cells relay messages through exosomes in kidney disease 10, 12, 13. To test whether exosomes also play a role in the above process, we treated BSA-HK2 cells with GW4869, an N-SMase inhibitor involved in inhibiting exosome release. Notably, exosome inhibition reversed BSA-HK2 cell-induced normal HK2 cellular injury (Figure 1D-F). Then, we isolated exosomes from HK2 cells, and the exosomes were identified by electron microscopy (Figure 1G), western blotting (Figure 1H) and nanoparticle tracking analysis (Figure 1I). Moreover, we treated normal HK2 cells with exosomes from BSA-HK2 cells (BSA-exos) and Control-HK2 cells (Control-exos). As expected, BSA-exos significantly induced recipient HK2 cellular injury (Figure 1J-L).

To explore the ability of BSA-exos to aggravate renal fibrosis during DKD, we used db/db mice, a mouse model of type 2 diabetes. Using IVIS Lumina live animal biophotonic imaging, we found that most exosomes were distributed in liver and renal tissue (Figure 1M), and more BSA-exos than Control-exos were observed in kidneys, suggesting that the injury message could be more easily disseminated between tubular cells. We then examined the role of BSA-exos in the progression of DKD. As shown in Figure 1O, the UACR increased in the BSA-exo injection group. We used Masson staining to assess renal interstitial fibrosis (Figure 1P) and examined the expression levels of fibrosis and inflammation markers by RT‒PCR and western blotting (Figure 1Q-R). The results revealed that BSA-HK2-exo injection promoted the progression of DKD. Furthermore, using immunofluorescence, we found that KIM-1 expression increased in mice that had been injected with BSA-HK2-exos (Figure 1S). These results suggest that BSA-HK2 cell-derived exosomes could induce normal HK2 cellular injury and accelerate DKD progression.

### SESN2 delays DKD progression

**Figure 2:**
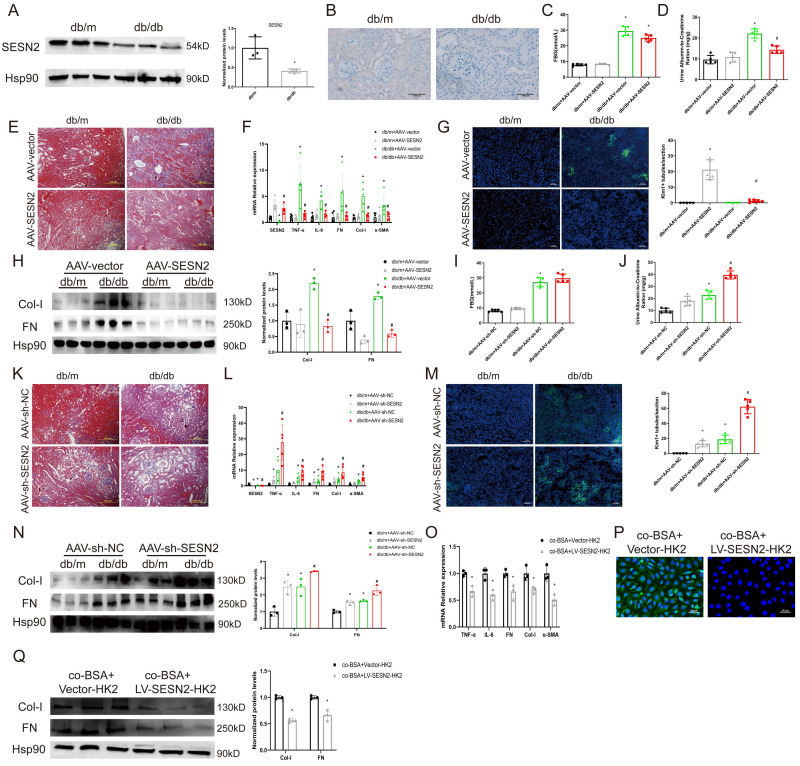
** SESN2 delays DKD progression.** (A) SESN2 was detected using western blot (B) and immunostaining (Scale bars: 100 μm.) in the kidney cortices of db/m and db/db mice. *p < 0.05 vs. the db/m group. Db/m and db/db mice were injected with AAV-vector or AAV-SESN2 via the tail vein: (C) Fasting blood glucose. (D) Urinary ACR levels. (E) Masson staining of kidney cortex. Scale bars: 100 μm. (F) mRNA expression levels of SESN2, TNF-α, IL-6, FN, Col-I and α-SMA (n=5). (G) Representative immunostaining images of KIM-1(n=5). Scale bars: 50 μm. (H) Protein expression levels of Col-I and FN (n=3). *p < 0.05 vs. the db/m+AAV-vector group, #*p* < 0.05 vs. the db/db+AAV-vector group. Db/m and db/db mice were injected with AAV-sh-NC or AAV-sh-SESN2 via the tail vein: (I) Fasting blood glucose. (J) Urinary ACR levels. (K) Masson staining of kidney cortex. Scale bars: 100 μm. (L) mRNA expression levels of SESN2, TNF-α, IL-6, FN, Col-I and α-SMA (n=5). (M) Representative immunostaining images of KIM-1(n=5). Scale bars: 50 μm. (N) Protein expression levels of Col-I and FN (n=3). *p < 0.05 vs. the db/m+AAV-sh-NC group, #*p* < 0.05 vs. the db/db+AAV-sh-NC group. HK-2 cells were co-cultured with BSA-HK2 cells stably overexpressing Vector (co-BSA+Vector-HK2) or SESN2 (co-BSA+LV-SESN2-HK2): (O) mRNA expression levels of TNF-α, IL-6, FN, Col-I and α-SMA (n=3). (P) Representative immunostaining images of KIM-1. Scale bars: 50 μm; (Q) Protein expression levels of Col-I and FN (n=3). *p < 0.05 vs. the co-BSA+Vector-HK2 group.

In our previous research, we found that SESN2 could alleviate BSA-induced HK2 cell injury 17. To further explore the role of SESN2 in DKD, we examined SESN2 expression in the renal cortex via both WB (Figure 2A) and immunohistochemistry (Figure 2B). We confirmed that SESN2 was downregulated in db/db mice compared with db/m mice. Next, we injected db/m or db/db mice with AAV-SESN2 or AAV-shSESN2 to overexpress or knock down endogenous SESN2 expression, respectively (Figures 2F, 2L and S1A-B). We found that, compared with those injected with AAV-vector, db/db mice injected with AAV-SESN2 had significantly reduced UACR, renal fibrosis and Kim-1 expression (Figure 2D-H). As expected, AAV-sh-SESN2 aggravated db/db mouse UACR, renal fibrosis and Kim-1 expression (Figure 2I-N). Hence, the results of these *in vivo* studies indicate that SESN2 could delay the progression of DKD and alleviate tubular injury.

Because SESN2 delays renal tubular injury, we wondered whether SESN2 could also disrupt the communication between injured and normal tubular epithelial cells. We therefore cocultured BSA treated SESN2 stably overexpressed HK2 cells (BSA+LV-SESN2-HK2) with normal HK2 cells. As expected, overexpression of SESN2 reduced the BSA+Vector-HK2 cell-induced expression of fibrosis markers, inflammatory cytokines and Kim-1 in normal HK2 cells (Figure 2O-Q). Because BSA-HK2 cell-derived exosomes can induce normal HK2 cellular injury, we speculated that SESN2 may improve BSA-HK2 cell-induced normal HK2 cellular injury by altering exosome release or exosome content.

### SESN2 inhibits exosome secretion from renal tubular epithelial cells

**Figure 3:**
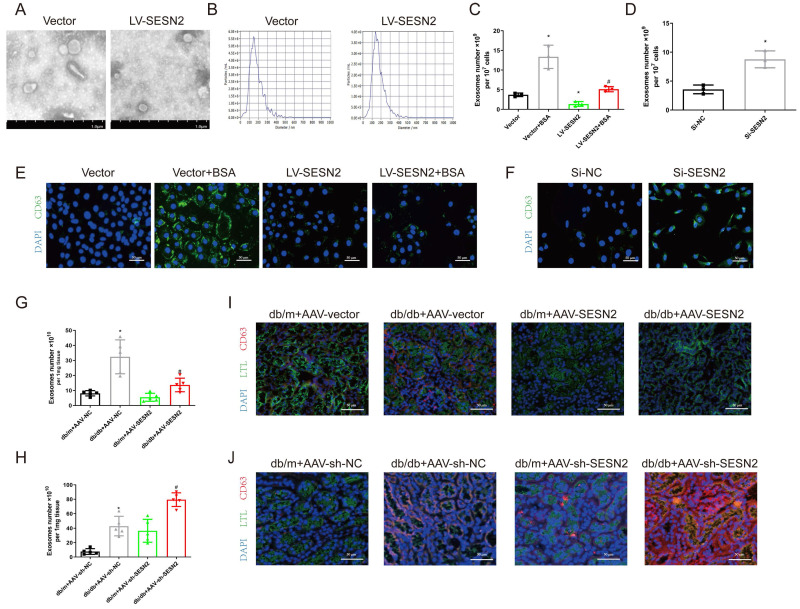
** SESN2 inhibits exosome secretion from renal tubular epithelial cells.** Exosomes were isolated from Vector or SESN2 stably overexpressed HK-2 cells and analyzed using (A) Representative TEM images (Scale bars: 1.0 μm) and (B) NTA analysis. (C) Exosome numbers from Vector or SESN2 stably overexpressing HK-2 cells treated with or without BSA (n=3). *p < 0.05 vs. the Vector group, #*p* < 0.05 vs. the Vector+BSA group. (D) Exosome numbers from Si-NC- or Si-SESN2-transfected HK-2 cells (n=3). *p < 0.05 vs. the Si-NC group. (E) Representative immunostaining images of CD63 in Vector or SESN2 stably overexpressing HK-2 cells treated with or without BSA. Scale bars: 50 μm. (F) Representative immunostaining images of CD63 in Si-NC- or Si-SESN2-transfected HK-2 cells. Scale bars: 50 μm. (G) Exosome numbers from db/m and db/db mice injected with AAV-vector or AAV-SESN2 via the tail vein (n=5). *p < 0.05 vs. the db/m+AAV-vector group, #*p* < 0.05 vs. the db/db+AAV-vector group. (H) Exosome numbers from db/m and db/db mice injected with AAV-sh-NC or AAV-sh-SESN2 via the tail vein (n=5). *p < 0.05 vs. the db/m+AAV-sh-NC group, #*p* < 0.05 vs. the db/db+AAV-sh-NC group. (I) Representative immunostaining images of LTL and CD63 in db/m and db/db mice injected with AAV-vector or AAV-SESN2 via the tail vein. Scale bars: 50 μm. (J) Representative immunostaining images of LTL and CD63 in db/m and db/db mice injected with AAV-sh-NC or AAV-sh-SESN2 via the tail vein. Scale bars: 50 μm.

To determine the role of SESN2 in exosome release, we isolated exosomes from HK2 cells stably expressing SESN2 and found that SESN2 had no effect on the morphology or size distribution of the exosomes (Figure 3A-B). To confirm the role of SESN2 in exosome release, we isolated exosomes and quantified them using an Exosomes ELISA complete kit and found that SESN2 overexpression reduced the number of exosomes by 61.6% in the supernatant compared with that in the Vector-BSA group (Figure 3C). siSESN2 also increased the number of exosomes in the supernatant of HK2 cells by 116% (Figure 3D). MVBs are the precursors of exosomes before secretion. Interestingly, immunostaining revealed that the expression of CD63, a marker of MVBs, was increased in HK-2 cells after BSA treatment and that this was reversed by SESN2 overexpression (Figure 3E). As expected, reducing SESN2 expression through transfection of siSESN2 also increased CD63 expression in HK2 cells (Figure 3F). Exosomes from the kidneys of mice were isolated using an exosome precipitation kit (Figure S2A-C), and we found that AAV-SESN2 reduced the exosome number by approximately 57.7% (Figure 3G), and AAV-sh-SESN2 increased the exosome number by approximately 85.3% (Figure 3H). We also costained for CD63 and the proximal tubule marker lotus tetragonolobus lectin (LTL) expression, and as shown in Figure 3I-J, CD63 expression was increased in the proximal tubules of db/db mice and was reversed by SESN2 overexpression; moreover, rAAV9-sh-SESN2 aggravated CD63 expression. Based on the results of these *in vivo* and *in vitro* studies, SESN2 could inhibit exosome release; thus, the reduced release of exosomes induced by SESN2 overexpression may be caused by a reduction in MVB accumulation in HK2 cells.

### SESN2 inhibits HK2 cell exosome release by promoting autophagic degradation

**Figure 4:**
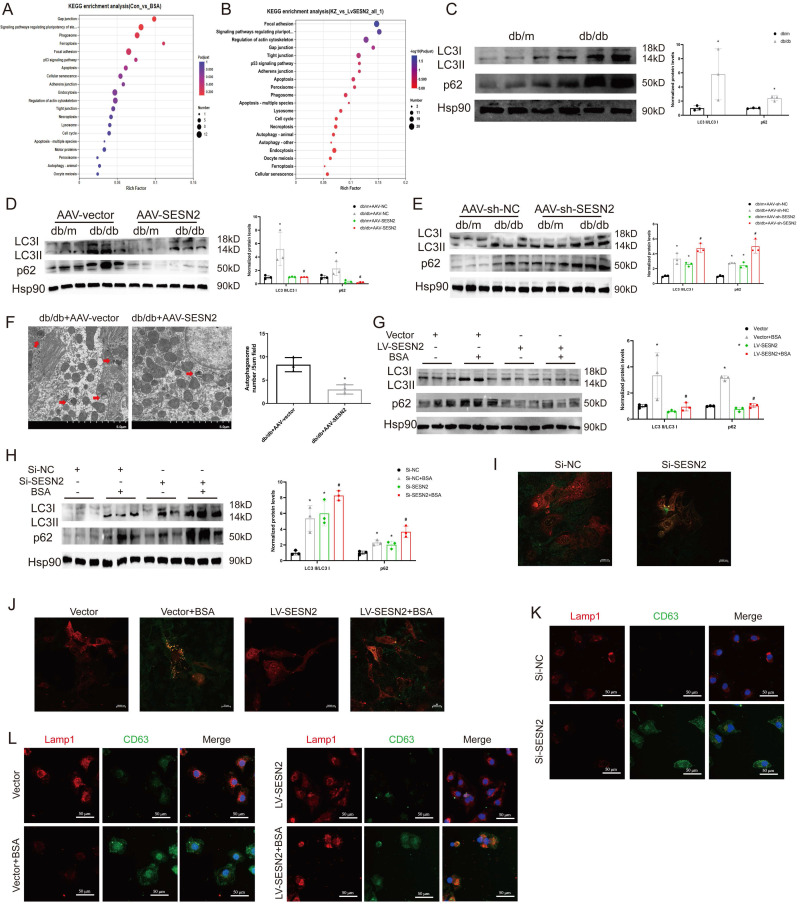
** SESN2 inhibits HK2 cell exosome release by promoting autophagic degradation.** (A-B) KEGG pathway analysis according to RNA-sequencing results. (C) Protein expression levels of LC3II:I and p62 in the kidney cortices of db/m and db/db mice (n=3). *p < 0.05 vs. the db/m group. (D) Protein expression levels of LC3II:I and p62 in the kidney cortices of db/m and db/db mice injected with AAV-vector or AAV-SESN2 via the tail vein. (n=3) . *p < 0.05 vs. the db/m+AAV-vector group, #*p* < 0.05 vs. the db/db+AAV-vector group. (E) Protein expression levels of LC3II:I and p62 in the kidney cortices of db/m and db/db mice injected with AAV-sh-NC or AAV-sh-SESN2 via the tail vein. (n=3). *p < 0.05 vs. the db/m+AAV-sh-NC group, #*p* < 0.05 vs. the db/db+AAV-sh-NC group. (F) Kidney cortex electron microscopic images of db/db mice injected with AAV-vector or AAV-SESN2 via the tail vein. The autophagosome (red arrows) numbers were quantified (n= 3). Scale bars: 5.0 μm. (G) Protein expression levels of LC3II:I and p62 in Vector or SESN2 stably overexpressing HK-2 cells treated with or without BSA (n=3). *p < 0.05 vs. the Vector group, #*p* < 0.05 vs. the Vector+BSA group. (H) Protein expression levels of LC3II:I and p62 in Si-NC- or Si-SESN2-transfected HK-2 cells treated with or without BSA (n=3). *p < 0.05 vs. the Si-NC group, #*p* < 0.05 vs. the Si-NC+BSA group. (I) Si-NC- or Si-SESN2-transfected HK-2 cells were infected with RFP-GFP-LC3-expressing adenovirus, and the fluorescence was detected by confocal microscopy. Scale bars: 2.0 μm. (J) Vector or SESN2 stably overexpressing HK-2 cells treated with or without BSA were infected with RFP-GFP-LC3-expressing adenovirus, and the fluorescence was detected by confocal microscopy. Scale bars: 2.0 μm. (K) Representative immunostaining images of CD63 and LAMP1 in Si-NC- or Si-SESN2-transfected HK-2 cells. Scale bars: 50 μm. (L) Representative immunostaining images of CD63 and LAMP1 in Vector or SESN2 stably overexpressing HK-2 cells treated with or without BSA. Scale bars: 50 μm.

To explore the potential mechanism through which SESN2 regulates exosome release, we performed RNA-seq on HK2 cells treated with BSA or overexpressed SESN2. A total of 867 differentially expressed genes (DEGs) were identified between the Control and the BSA group, and 3717 DEGs were identified between the LV-SESN2 subgroup and the Vector group (Sup Fig. 3A-B). Genes associated with the autophagy-lysosome pathway were changed between both different groups (Figure 4A-B). We also performed intersection analysis on the two RNA-seq datasets and observed persistent enrichment of the autophagy-lysosome pathway even after overlap filtering (Sup Fig. 3C-D), suggesting that the autophagy-lysosome pathway is a consistent and potentially critical pathway affected by SESN2 in the context of BSA treatment. Several studies have reported that impaired autophagic degradation inhibits MVB degradation through the autophagy‒lysosome pathway via fusion with the plasma membrane and secretion as exosomes 22, 24. We therefore investigated whether SESN2 inhibits exosome secretion by regulating the autophagy-lysosome pathway.

First, we measured the ratio of LC3BII:I, which reflects the number of autophagosomes, and p62 expression, which negatively correlates with autophagy activity, in db/db mice. As shown in Figure 4C, the level of LC3BII:I and the accumulation of p62 increased in the renal cortices of db/db mice, which indicated that autophagosome degradation decreased in the renal cortices during DKD. After treatment of mice with AAV9-SESN2, the ratio of LC3BII:I and the accumulation of p62 decreased (Figure 4D), whereas SESN2 knockdown further prevented autophagic degradation (Figure 4E). Then, we used TEM to observe autophagosomes in the mouse renal cortex and found that SESN2 overexpression alleviated the retention of autophagosomes in db/db mice (Figure 4F). We also measured the ratio of LC3BII:I and p62 expression in HK2 cells. As expected, the LC3BII:I ratio and p62 accumulation increased in the siSESN2 group (Figure 4H) but decreased in the SESN2-overexpressing group (Figure 4G), indicating that SESN2 promoted the degradation of autophagosomes.

Then, we transfected HK2 cells with an RFP-GFP-LC3-expressing adenovirus to confirm the role of SESN2 in autophagic flux. When GFP fluorescence is quenched after fusion with lysosomes and autophagosomes are degraded, the signal appears red; when autophagic flux is disrupted, the nonacid autophagosomes appear yellow because of the overlay of green and red fluorescence. Confocal microscopy results showed that SESN2 inhibition increased the presence of yellow puncta (Figure 4I), whereas SESN2 overexpression showed the opposite effect (Figure 4J). Therefore, these results show that SESN2 alleviates autophagic flux in the renal tubular cells during DKD. Next, we observed the degradation of MVBs by lysosomes through determination of the colocalization of CD63 and LAMP1 expression. As shown in Figure 4K-L, si-SESN2 suppressed the colocalization of CD63 and LAMP1, whereas SESN2 overexpression had the opposite effect. Taken together, these results suggest that SESN2 inhibits exosome release by blocking autophagic degradation.

### SESN2 binds to Rab-7a to enhance its stability and prevent Rab-7a ubiquitination

**Figure 5:**
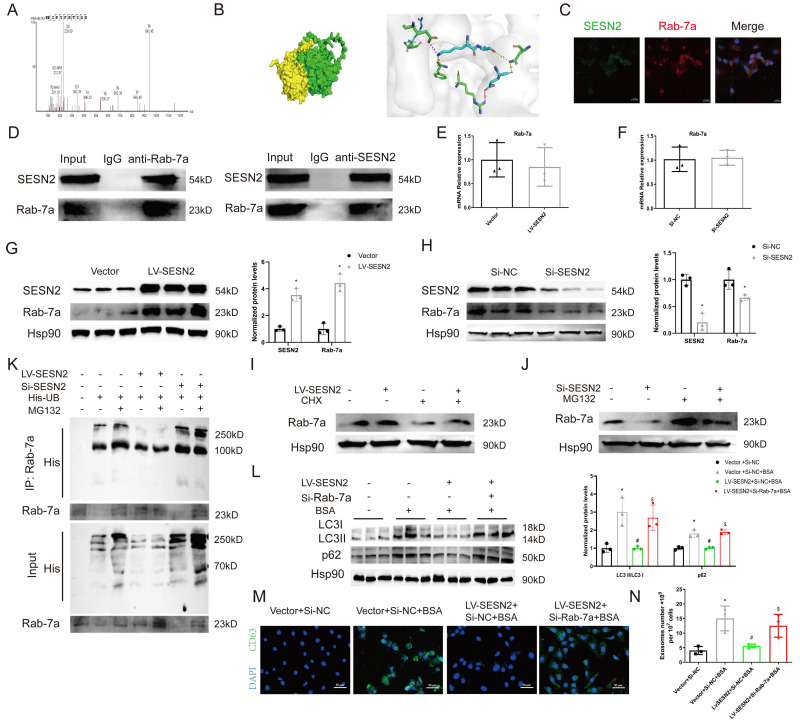
** SESN2 binds with Rab-7a to enhance its stability and prevent Rab-7a ubiquitination.** (A) Cell lysates were pulled down from Vector or SESN2 stably overexpressing HK-2 cells using a SESN2 antibody, and Rab-7a levels were found to be increased in the LV-SESN2 group by mass spectrometry. (B) Diagram of a protein-protein docking model and interfacing residues between SESN2 and Rab-7a proteins (green: Rab-7a; yellow: SESN2). (C) Confocal microscopy images of the co-localization of SESN2 and Rab-7a in HK-2 cells. Scale bars: 20 μm. (D) Co-immunoprecipitation showing the interaction between SESN2 and Rab-7a in HK-2 cells. (E) mRNA expression levels of Rab-7a were measured in Vector or SESN2 stably overexpressing HK-2 cells (n=3). (F) mRNA expression levels of Rab-7a were measured in Si-NC- or Si-SESN2-transfected HK-2 cells (n=3). (G) Protein expression levels of Rab-7a and SESN2 were measured in Vector or SESN2 stably overexpressing HK-2 cells (n=3). *p < 0.05 vs. the Vector group. (H) Protein expression levels of Rab-7a and SESN2 were measured in Si-NC- or Si-SESN2-transfected HK-2 cells (n=3). *p < 0.05 vs. the Vector group. (I) Vector or SESN2 stably overexpressing HK-2 cells were treated with 50 μg/mL cycloheximide, and cell lysates were subjected to Western blotting analysis of Rab-7a. (J) Si-NC- or Si-SESN2-transfected HK-2 cells were treated with 10 μM of MG132, and cell lysates were subjected to Western blotting analysis of Rab-7a. (K) HK-2 cells were transfected with His-Ub, LV-SESN2 or Si-SESN2 and then treated with MG132. The ubiquitylation level of Rab-7a was detected using an anti-His antibody. Vector or SESN2 stably overexpressing HK-2 cells treated with or without BSA were infected with Si-NC or Si-Rab-7a: (L) Protein expression levels of LC3II:I and p62 (n=3). (M) Representative immunostaining images of CD63. Scale bars: 50 μm. (N) Total exosome proteins. *p < 0.05 vs. the Vector+Si-NC group, #*p* < 0.05 vs. the Vector+Si-NC+BSA group, ^$^*p* < 0.05 vs. the LV-SESN2+Si-NC+BSA group.

To identify the potential molecular mechanisms through which SESN2 regulates autophagic degradation, whole-cell protein lysates from SESN2-overexpressing cells were subjected to IP-MS with IgG and SESN2 antibodies. We verified that Rab-7a (Figure 5A), a key protein that promotes the fusion of autophagosomes and lysosomes, was a putative SESN2-binding protein. We also performed protein-protein docking to verify the relationship between SESN2 and Rab-7a (Figure 5B and Table S1). Immunofluorescence confirmed the colocalization between SESN2 and Rab-7a (Figure 5C), and the results of co-IP further confirmed the interaction between SESN2 and Rab-7a (Figure 5D).

To further explore the relationship between SESN2 and Rab-7a, we measured the expression of Rab-7a after SESN2 overexpression or knockdown. The results showed that the Rab-7a protein level decreased when SESN2 was knocked down and increased when SESN2 was overexpressed (Figure 5G, H), whereas Rab-7a mRNA levels did not change (Figure 5E, F). These results indicate that SESN2 influences the protein stability of Rab-7a, but the regulatory mechanism involved is unknown. As Rab-7a stability has been reported to be regulated by the ubiquitin‒proteasome pathway 25, 26, we wondered whether SESN2 also stabilized Rab-7a through this pathway. As expected, in the presence of CHX, Rab-7a degradation was greatly reduced after SESN2 overexpression (Figure 5I), whereas the proteasome inhibitor MG132 prevented siSESN2-induced Rab-7a degradation (Figure 5J). Moreover, a ubiquitination assay showed that Rab-7a was ubiquitinated and that its level was increased in the SESN2 knockdown group but decreased in the SESN2 overexpression group (Figure 5K). These observations reveal that SESN2 binds to Rab-7a to enhance its stability and prevent its ubiquitin-mediated degradation.

Subsequently, we transfected siRab-7a into SESN2-overexpressing cells. The BSA-induced increase in autophagosome retention was alleviated by SESN2 overexpression but was completely blocked in the siRab-7a group (Figure 5L). Moreover, the decreases in CD63 expression (Figure 5M) and exosome release (Figure 5N) in SESN2-overexpressing cells were reversed by the addition of siRab-7a. These results indicate that SESN2 regulates autophagic degradation and exosome release through Rab-7a.

### SESN2 promotes MVB degradation through regulation of TFEB subcellular localization

**Figure 6:**
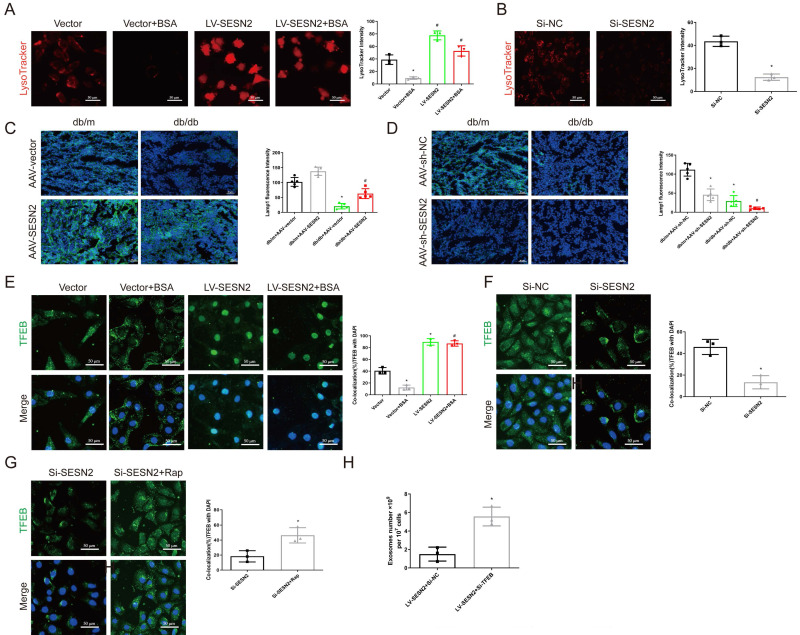
** SESN2 promotes MVB degradation through the regulation of TFEB subcellular localization.** (A) Representative fluorescence microscopy images of Vector or SESN2 stably overexpressing HK-2 cells treated with or without BSA stained with LysoTracker probe (n=3). Scale bars: 50 μm. *p < 0.05 vs. the Vector group, #*p* < 0.05 vs. the Vector+BSA group. (B) Representative fluorescence microscopy images of Si-NC- or Si-SESN2-transfected HK-2 cells stained with LysoTracker probe (n=3). Scale bars: 50 μm. *p < 0.05 vs. the Si-NC group. (C) Representative immunostaining images of Lamp1 (n=5) in the kidney cortices of db/m and db/db mice injected with AAV-vector or AAV-SESN2 via the tail vein. Scale bars: 50 μm. *p < 0.05 vs. the db/m+AAV-vector group, #*p* < 0.05 vs. the db/db+AAV-vector group. (D) Representative immunostaining images of Lamp1 (n=5) in the kidney cortices of db/m and db/db mice injected with AAV-sh-NC or AAV-sh-SESN2 via the tail vein. Scale bars: 50 μm.*p < 0.05 vs. the db/m+AAV-sh-NC group, #*p* < 0.05 vs. the db/db+AAV-sh-NC group. (E) Representative immunostaining images of TFEB in Vector or SESN2 stably overexpressing HK-2 cells treated with or without BSA. Scale bars: 50 μm. TFEB and DAPI colocalization was analyzed (n=3). *p < 0.05 vs. the Vector group, #*p* < 0.05 vs. the Vector+BSA group. (F) Representative immunostaining images of TFEB in Si-NC- or Si-SESN2-transfected HK-2 cells. Scale bars: 50 μm. TFEB and DAPI colocalization was analyzed (n=3). *p < 0.05 vs. the Si-NC group. (G) Representative immunostaining images of TFEB in Si-SESN2-transfected HK-2 cells treated with or without rapamycin. Scale bars: 50 μm. TFEB and DAPI colocalization was analyzed (n=3). *p < 0.05 vs. the Si-SESN2 group. (H) Exosome numbers from SESN2 stably overexpressing HK-2 cells transfected with Si-NC or Si-TFEB (n=3). *p < 0.05 vs. the Vector group, #*p* < 0.05 vs. the Vector+BSA group.

Several studies have reported that proximal tubular lysosomal function is defective in DKD 27, as the function of lysosomes is also a key factor in regulating MVB degradation, we propose that SESN2 may also promote MVB degradation through improving lysosomal function. Using LysoTracker staining, we found that SESN2 overexpression reversed the decrease in the number of acidic lysosomes compared with that in the BSA group (Figure 6A), whereas a decrease in the number of acidic lysosomes was found in the siSESN2 group (Figure 6B). Our data further demonstrated that BSA exposure increased lysosomal pH and increased the colocalization between galectin-3 and LAMP1, while SESN2 overexpression reserved this effect. SiSESN2 showed the same effect as BSA, suggesting that SESN2 could alleviate lysosomal stress (Sup Fig. 4A-D). Moreover, in db/db mice, the expression of the lysosome marker LAMP1 increased after AAV9-SESN2 injection (Figure 6C) but further decreased in the db/db+AAV9-sh-SESN2 group (Figure 6D). Taken together, these data indicate that SESN2 could improve lysosomal biogenesis and function. TFEB is the key transcription factor that participates in the regulation of lysosome biogenesis- and function-related genes and is reportedly phosphorylated by mTOR to inhibit its nuclear translocation and functional performance 28. To determine whether SESN2 regulates the subcellular localization of TFEB, HK2 cells were analyzed by immunofluorescence using an anti-TFEB antibody. As shown in Figure 6E, BSA treatment reduced TFEB expression in the cytoplasm, while SESN2 overexpression induced the translocation of TFEB into the nucleus. We also observed cytoplasmic retention of TFEB in the siSESN2 group (Figure 6F). To further confirm that SESN2 regulates subcellular localization of TFEB through the mTOR pathway, we treated the siSESN2 group with rapamycin, which inhibits mTOR activity. As expected, rapamycin reversed siSESN2-induced TFEB retention in the cytoplasm (Figure 6G), which indicated that SESN2 regulated TFEB subcellular localization through mTOR.

Next, we investigated whether SESN2 regulates the secretion of exosomes through TFEB by transfecting SESN2-overexpressing cells with siTFEB. We found that the reduction in the secretion of exosomes induced by SESN2 overexpression was also reversed by siTFEB administration (Figure 6H). These findings reveal that SESN2 alleviates lysosomal function through TFEB, thus promoting MVB and lysosome fusion and further reducing exosome secretion. Lysosomal exocytosis, which is mediated by the fusion of lysosomes with the plasma membrane, with subsequent release of the lysosomal cargo, is essential for preserving cellular homeostasis 29. A previous study reported that TFEB promoted lysosomal exocytosis 30. The use of an anti-LAMP1 antibody demonstrated that SESN2 promoted LAMP1 exposure on the plasma membrane (Sup Fig. 4E-F), which indicated that SESN2 promoted lysosomal exocytosis.

### SESN2-overexpressing HK2 cell-derived exosomes alleviate tubular injury

**Figure 7:**
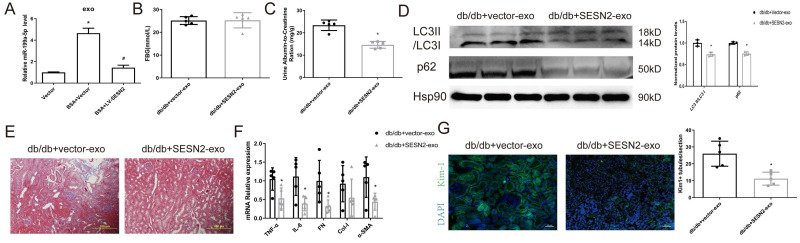
** SESN2-overexpressing HK2 cell-derived exosomes alleviate tubular injury.** (A) Vector or SESN2 stably overexpressing HK-2 cells were treated with or without BSA, and miR-199a-5p expression in HK-2 cell-derived exosomes was measured by RT-PCR. Db/db mice were injected with Vector or SESN2 stably overexpressing HK-2 cell-derived exosomes (db/db+Vector-exos and db/db+SESN2-exos). (B) Fasting blood glucose. (C) Urinary ACR levels. (D) Protein expression levels of LC3II:I and p62. (E) Masson staining of kidney cortex. Scale bars: 100 μm. (F) mRNA expression levels of TNF-α, IL-6, FN, Col-I and α-SMA (n=5). (G) Representative immunostaining images of KIM-1 (n=5). Scale bars: 50 μm. *p < 0.05 vs. the db/db+Vector-exos group.

We then examined whether SESN2 improves BSA-HK2 cell-induced normal HK2 cellular injury by changing the content of exosomes. We previously investigated the expression of miR-199a, which increased significantly in HSA-induced HK2 cell-derived exosomes, and reported that miR-199a participated in renal tubular injury 12. We found that miR-199a expression increased after BSA treatment but decreased after SESN2 was overexpressed in exosomes from HK2 cells, which may indicate that SESN2 expression changes not only exosome numbers but also exosome content (Figure 7A). Furthermore, we explored whether SESN2-overexpressing cell-derived exosomes (SESN2-exos) also could alleviate DKD progression *in vivo*. As shown in Figure 7B-G, the UACR decreased in the SESN2-exo injection group, while renal fibrosis, impaired autophagic degradation and Kim-1 expression were alleviated in the mice injected with SESN2-exos. These results suggested that SESN2-overexpressing HK2 cells could also delay DKD progression through secreted exosomes.

## Discussion

Renal tubular injury is the major feature underlying renal interstitial fibrosis and is linked with DKD outcomes. Owing to their high energy requirement, tubular epithelial cells are the initial reactors after damage, and they also expand this damage through intercellular cross talk 31. Albuminuria is one of the major causes of renal tubular injury in DKD patients. In this study, we found that BSA not only directly caused tubular injury but also promoted injured tubules to “infect” normal tubules through exosomes. Through *in vivo* and *in vitro* experiments, we confirmed that SESN2 could inhibit this “infection” through the reduction of exosome release by promoting autophagic degradation; Rab-7a-mediated autophagosome and lysosome fusion and TFEB-mediated lysosomal function repair may play roles in this process.

Renal tubules and tubulointerstitial tissue account for approximately 90% of the renal parenchyma, and dysfunction of these tissues plays a crucial role in DKD progression, especially in albuminuria-induced renal injury 32. Several studies have reported that renal tubular cell injury signals can spread intercellularly through exosomes, thus inducing tubulointerstitial fibrosis. Tsai *et al.* reported that high glucose-induced HK2 cells could induce normal cells to undergo epithelial-mesenchymal transition (EMT) through exosomal fibulin-1^8^. Another study revealed that TGF-β1-induced renal tubular cell-derived exosomes could promote the EMT of normal cells through miR-21^6^. In our study, we found that BSA-induced HK2 cell-derived exosomes promoted not only normal cell injury but also DKD progression. As current approaches to reduce albuminuria still cannot prevent the progression of renal tubule interstitial fibrosis, we propose that injured tubular cell-derived exosomes may play a key role in this process and that blocking the crosstalk between tubular cells may serve as a new therapeutic method.

Our previous research revealed that SESN2 could alleviate HSA-induced HK2 cell EMT and ER stress 17. Bian *et al.* found that SESN2 overexpression suppressed mesangial cell proliferation and attenuated renal fibrosis by modulating the Hippo signaling pathway during DKD 33. In this study, we found that SESN2 overexpression improved BSA-HK2 cell-induced Normo-HK2 cellular injury through reducing the release of BSA-HK2 cell-derived exosomes. RNA-seq revealed that SESN2 may regulate exosome secretion through the autophagy-lysosome pathway. Exosomes are secreted during the fusion of MVBs with the cell surface; before this step, most MVBs are destined to fuse with lysosomes for degradation. Moreover, the fusion of MVBs with autophagosomes influences their degradation; thus, reducing autophagosome degradation also promotes exosome release 15. Several studies have reported that overloading proteins impairs autophagy and leads to lysosomal dysfunction in DKD 34, 35. SESN2 is a positive modulator of autophagy and has been reported to induce light chain 3 expression and formation of autophagosomes in renal tubular (NRK-52E) cells 36. In the present study, we demonstrated that SESN2 promoted autophagosomal degradation, which was accompanied by decreased CD63 accumulation in HK2 cells and decreased exosome release. In addition, we observed that SESN2 alleviated lysosome dysfunction and promoted the fusion of MVBs with lysosomes. Taken together, these findings suggest that SESN2 alleviates albuminuria-induced autophagic-lysosomal pathway disruption and lysosomal dysfunction, thus promoting autophagic degradation and reducing exosome release.

Autophagosome and lysosome fusion is the final step of autophagy and requires the coordination of SNAREs, small GTPases, tethering factors, and other proteins 37. The exploration of the downstream target of SESN2 in the regulation of autophagosome and lysosome fusion led to the identification of Rab-7a. Rab-7a is a late endosomal small GTPase implicated in late endosome/lysosome fusion, and its two effectors, PLEKHM1 and EPG5, are responsible for autophagosome-lysosome fusion 38. We found that the Rab-7a protein level increased when SESN2 was overexpressed. In addition, by using IP and immunofluorescence, we demonstrated the physical interaction and colocalization of SESN2 with Rab-7a. The ubiquitin‒proteasome system is a key mechanism for protein degradation. It has been reported that Rab-7a has several ubiquitination sites and that its ubiquitination influences its expression levels and activity 25, 26. Thus, we proposed that siSESN2 might promote Rab-7a degradation through the ubiquitination pathway and used a co-IP assay to show that siSESN2 promoted Rab-7a ubiquitination. However, in the present study, we did not further explore the ubiquitination sites of Rab-7a, and thus, further studies are needed to explore the underlying mechanisms involved. As expected, the knockdown of Rab-7a with a siRNA reversed the SESN2-induced autophagosome-lysosome fusion. In this regard, we found that SESN2 regulated exosome release through Rab-7a-induced autophagosome-lysosome fusion.

Several previous studies have indicated that albuminuria can damage renal tubular cells by inducing lysosomal dysfunction 34, 35. In this study, we observed that SESN2 alleviated BSA-induced lysosomal dysfunction. Transcription factor EB (TFEB) can directly bind to the coordinated lysosomal expression and regulation (CLEAR) gene network and mediate lysosome-related gene transcription 39. The activity of TFEB is determined by its subcellular localization, and mTORC1 has been reported to promote TFEB phosphorylation, thus leading to its cytoplasmic retention and inactivation 28. SESN2 could inhibit the activity of mTORC1 40, and the results of our study showed that SESN2 markedly increased TFEB nuclear translocation through mTORC1. Collectively, these findings shed new light on the protective effect of SESN2 on DKD through the targeting of TFEB and lysosomes.

In summary, we showed that the upregulation of SESN2 alleviated the BSA-induced reduction in Rab-7a levels and TFEB cytoplasmic retention in injured HK2 cells, which promoted the fusion of autophagosomes and lysosomes and further inhibited the secretion of injury information-containing exosomes. Moreover, SESN2-overexpressing renal tubular cell-derived exosomes alleviated DKD progression. These findings provide insights into the underlying molecular mechanisms involved as well as evidence that SESN2 can prevent the spread of injury information between tubular cells and may constitute a promising strategy for preventing and treating DKD.

## Methods

### Animal model

Seven-week-old db/db mice on a C57BL/6J background and their age-matched normal littermates (db/m mice) were purchased from the Model Animal Research Center of Nanjing University. All mice were housed in specific pathogen-free conditions with free access to water and food. To study the effects of exosomes, twenty-week-old db/db mice were randomly divided into four groups: the db/db+Control-exo group, the db/db+BSA-exo group, the db/db+Vector-exo group and the db/db+SESN2-exo group. Sterile exosomes were collected from HK-2 cells, quantified using the BCA method and then intravenously injected three times a week (100 µg/mouse) for four weeks. To investigate the influence of SESN2 on DKD, both db/db mice (n=20) and db/m mice (n=20) were randomized into 4 groups: AAV-vector, AAV-SESN2, AAV-sh-NC, and AAV-sh-SESN2. Eight-week-old mice were administered 0.1 ml of solution containing 10^11^ genome copies (GCs)/animal infective units of recombinant adeno-associated virus (AAV; GeneChem, Shanghai, China) via the tail vein, and RT‒PCR and western blotting were used to confirm the efficacy of the treatments. All the mice were fasted for more than 8 h to measure blood glucose levels; urine samples were collected; and the mice were euthanized at 24 weeks. The animal experiments were approved by the Institutional Animal Care and Use Committee of the Laboratory Animal Center at Southern Medical University (certificate number: L2018022).

### Serum and urine biochemistry assays

Blood glucose was measured by an ELISA. Urinary concentrations of albumin and creatinine were measured using a Mouse Albumin ELISA Kit (Bethyl Laboratories, Inc., Montgomery, TX) and a Quanti Chrom Creatinine Assay Kit (BioAssay Systems, USA), respectively. The urinary albumin-to-creatinine ratio (UACR) was calculated as the albumin/creatinine ratio (mg/g).

### Renal histopathology

For histological analysis, the renal cortex was fixed with paraformaldehyde, embedded in paraffin, and then sliced into 4 µm sections. Then, the sections were stained with Masson's trichrome and PAS according to standard protocols. For SESN2 staining, sections were incubated with an anti-SESN2 antibody (66297-1-Ig, Proteintech, Rosemont, USA) overnight at 4 °C, followed by incubation with secondary antibodies. Sections were observed and photographed using an Olympus B upright light microscope (Olympus, Japan).

### Cell culture and treatment

Human proximal tubular epithelial (HK-2) cells were obtained from the Shanghai Cell Bank of the Chinese Academy of Sciences (China), and were cultured in Roswell Park Memorial Institute (RPMI) 1640 medium supplemented with 10% fetal bovine serum at 37 °C in a 5% CO2 atmosphere. In some experiments, HK-2 cells were treated with 10 mg/ml BSA for 48 h after serum starvation. Rapamycin (MedChemExpress, Shanghai, China) was used to inhibit the activity of mTOR at a concentration of 100 mmol/L.

To study the interaction between HK-2 cells, Transwell coculture systems (Corning, MA, USA) were used. Donor HK-2 cells were treated with BSA for 48 h, the serum medium was replaced, and the cells were then cocultured with Normo-HK2 cells. In some experiments, donor HK-2 cells were pretreated with 10 μM GW4869 for 24 h before coculture. To study the role of exosomes, recipient cells were cocultured with 30 µg/ml exosomes from donor cells for 48 h.

Lentivirus-loaded SESN2-overexpressing plasmids labelled with GFP (RiboBio, Guangzhou, China) and the corresponding control vector were transfected into HK-2 cells in the presence of puromycin (MedChemExpress, Shanghai, China) for 48 h. Two rounds of infection were performed. After infection, the surviving cells were selected and collected for an additional 24 h for further experiments.

For siRNA-mediated knockdown, 50 nM SESN2 siRNA, Rab-7a siRNA or TFEB siRNA was transfected into HK-2 cells. Lipofectamine 3000 transfection reagent (Invitrogen, Carlsbad, USA) was used for transfection according to the manufacturer's instructions.

### Exosome isolation and quantification

Exosomes from conditioned media were isolated using differential centrifugation methods as follows: Before the supernatants were collected, the medium was changed to serum-free medium, and the cells were cultured for 48 h. Next supernatants were collected and centrifuged at 2,000 × *g* for 30 min to remove cell debris, then supernatants were collected and centrifuged at 10,000 × *g* for 30 min and filtrated through a 0.22-mm pore filters, then ultracentrifugation at 100,000 × *g* for 90 min. The pellets were resuspended in PBS and ultracentrifugation at 100,000 × *g* for another 90 min. Finally, pellets were dissolved in PBS for further research. For mouse renal exosome isolation, 50 mg of renal cortex tissue was excised, digested with DMEM containing collagenase IV at 37 °C for 2 h, and neutralized with exosome-free FBS. The homogenate was centrifuged at 200 × *g* for 30 min and 3,000 × *g* for 15 min, after which 1:5 volume of ExoQuick-TC (System Biosciences, USA) was added. The mixture was mixed well and further incubated at 4 °C overnight. The next day, the mixture was centrifuged at 1,300 × g for 30 min, and finally the pellets were dissolved in PBS and filtered through 0.22-mm pore filters. Exosome numbers were quantified using a CD63 ExoELISA ULTRA kit (SBI, USA).

### Transmission electron microscopy (TEM)

For autophagosome detection, fresh renal cortices were sliced into 1 mm^3^ pieces, fixed in ice-cold 2.5% glutaraldehyde, and were observed and imaged via TEM (HT7800, Hitachi, Tokyo, Japan). For exosome viewing, exosomes were loaded on 200-mesh nickel grids for 1 min and stained with 2% phosphotungstic acid for 1 min. After removing the phosphotungstic acid, the cells were air dried. The morphology was also observed using TEM (HT7800, Hitachi, Tokyo, Japan).

### Nanoparticle tracking analysis (NTA)

The concentration and size distribution of the exosomes were analyzed by means of a ZetaView Particle Metrix (Meerbusch, Germany). Briefly, exosomes were diluted in 1× PBS and then injected into the sample chamber. The data were analyzed with the corresponding software ZetaView 8.05.14.

### *In vivo* biodistribution of sEVs

Did (5 µl/ml, KGMP0025, KeyGEN BioTECH, China)-labelled exosomes were injected into db/db mice as previously reported. An *in vivo* imaging system (IVIS) (Ami HTX, Spectral Instruments Imaging, USA) was used to observe the exosome distribution in the mice.

### RNA extraction and qRT‒PCR

Total RNA was extracted from the samples using TRIzol reagent (Invitrogen) according to the manufacturer's protocol. After using the 5× PrimeScript RT Master Mix Kit (Yeasen Biotech, Shanghai) for reverse transcription, the SYBR Premix Ex Taq™ Kit (Yeasen Biotech, Shanghai) and a QuantStudio 6 Flex Real-Time PCR System (Thermo Scientific, USA) were used for real-time PCR. The expression levels of the target genes were normalized to that of β-actin and calculated using the 2^-ΔΔCT^ method. The sequences of primers used are listed in Supplementary Table S2.

### Western blot analysis

The renal cortex, HK-2 cells and exosomes were lysed using RIPA lysis buffer (Beyotime, China), and the protein concentration was measured using a BCA protein assay kit (Takara, China). Then, equal amounts of proteins from the renal cortex and HK-2 cells and proteins from equal numbers of cell-derived exosomes were loaded for SDS-PAGE and transferred onto PVDF membranes (Merck, USA). The membranes were incubated overnight with 5% BSA in TBS supplemented with primary antibodies against FN (15613-1-AP; Proteintech, Rosemont, USA), Col-I (14695-1-AP; Proteintech, Rosemont, USA), SESN2 (66297-1-Ig; Proteintech, Rosemont, USA), p62 (18420-1-AP; Proteintech, Rosemont, USA), LC3 (14600-1-AP; Proteintech, Rosemont, USA), Rab-7a (55469-1-AP; Proteintech, Rosemont, USA), CD63 (A5271; ABclonal, USA), and TSG101 (A2216; ABclonal, USA). Further corresponding secondary antibodies were applied, and protein bands were detected using an enhanced chemiluminescence system (GelView 6000 Pro, Guang Zhou, China). Protein expression levels were analyzed with Quantity One software (Bio-Rad, CA, USA) and normalized to the level of HSP90 (13171-1-AP; Proteintech, Rosemont, USA).

### Coimmunoprecipitation and mass spectrometry (MS)

Protein A magnetic beads (Thermo Fisher Scientific, Waltham, MA, USA) were incubated with an anti-SESN2 antibody (66297-1-Ig; Proteintech, Rosemont, USA) or an anti-Rab-7a antibody (55469-1-AP; Proteintech, Rosemont, USA) for 30 min and then incubated with the cell lysates overnight at 4 °C. Then, the magnetic beads were washed and 1 × SDS‒PAGE buffer was added to the purified proteins, which were subsequently boiled for 10 min at 95 °C. Captured proteins were separated via SDS‒PAGE and visualized via Coomassie blue staining or western blotting. For MS, the gels were stained with Coomassie blue (Beyotime, China), and MS was performed by Wininnovate Bio (Shenzhen, China). The MS/MS data were analyzed for protein identification and quantification using PEAKS Studio 8.5.

### Protein-protein docking analysis

The protein structural domains of SESN2 and Rab-7a were downloaded from the Protein Data Bank (PDB) database (http://www.rcsb.org/). The protein-protein docking between these two proteins was analyzed using GRAMM-X (http://gramm.compbio.ku.edu/). PDBePISA (https://www.ebi.ac.uk/pdbe/pisa/) was used to explore protein-protein interactions, and PyMOL (Version 2.4) was used for visualization and analysis.

### *In vivo* ubiquitination assay

HK-2 cells were transiently transfected with a plasmid containing His-Ub in the presence or absence of SESN2 overexpression plasmid or siSESN2 with or without MG132. Then, the cells were lysed and immunoprecipitated by using an anti-Rab-7a antibody (55469-1-AP; Proteintech, Rosemont, USA). Finally, the proteins were subjected to western blotting with an anti-His antibody (66005-1-Ig; Proteintech, Rosemont, USA) or an anti-Rab-7a antibody (55469-1-AP; Proteintech, Rosemont, USA) to detect Rab-7a ubiquitination.

### LysoTracker red or LysoSensor green DND-189 staining

Before cell harvest, the cellular supernatant was removed, and the cells were washed with PBS. LysoTracker Red (50 nM; Yeasen, China) or LysoSensor Green DND-189 (1 μM; Yeasen, China) was added to the culture medium, after which the cells were incubated at 37 °C for 30 min in the dark. Then, the cells were washed twice with PBS. Images were captured using an upright fluorescence microscope (Imager D2, Zeiss, Germany).

### Immunofluorescence staining

Mouse renal cortex frozen sections were thawed and washed with PBS. HK-2 cells were fixed in 4% paraformaldehyde and permeabilized with 0.1% Triton X-100. After being blocked with 5% BSA for 30 min at room temperature, the sections were incubated with the following primary antibodies: anti-CD63 (25682-1-AP; Proteintech; 1:150), anti-Kim-1 (A2831; ABclonal, USA), anti-TFEB (13372-1-AP; Proteintech; 1:150), anti-LAMP1 (WL02419; Wanleibio, China; 1:150), and anti-GAL3 (60207-1; Proteintech). After washing, the sections were incubated with secondary antibodies for 1 h. Nuclei were counterstained with DAPI. All images were obtained by an upright fluorescence microscope (Imager D2, Zeiss, Germany).

### RNA sequence analysis

Total RNA was extracted from HK-2 cells (n=3), BSA treated HK-2 cells (n=3), stable SESN2-overexpressing HK-2 cells (LV-SESN2) (n=3) and the corresponding control vector-overexpressing HK-2 cells (vector) (n=3) using TRIzol reagent (Invitrogen). The purity and concentration of RNA were assessed using a NanoDrop2000, and the integrity of the RNA was assessed by an Agilent 5300 spectrophotometer. Then, the RNA library was constructed, and RNA sequencing was performed using an Illumina NovaSeq Xplus platform at Majorbio Biotech (Shanghai, China). All the data were analyzed on the Majorbio Cloud Platform (https://cloud.majorbio.com/).

### Autophagic flux evaluation

HK-2 cells were transfected with an adenovirus expressing the mCherry-GFP-LC3B fusion protein at a multiplicity of infection (MOI) of 20 for 24 h. Then, the cellular supernatant was removed and changed to fresh complete culture medium for another 48 h. Finally, mCherry and GFP fluorescence was observed using a Zeiss LSM 980 microscope (Zeiss, Germany), and the number of puncta was calculated using ImageJ.

### Statistical analysis

All the data were obtained from at least three independent experiments and analyzed using GraphPad Prism software (version 9; GraphPad, USA). The results are presented as the mean ± SEM, and Student's t test or one-way ANOVA was used to analyze the results. A P value <0.05 was considered to indicate statistical significance.

## Supplementary Material

Supplementary figures and tables.

## References

[1] Cheng HT, Xu X, Lim PS, Hung KY (2021). Worldwide Epidemiology of Diabetes-Related End-Stage Renal Disease, 2000-2015. Diabetes care.

[2] Pieber TR, Bode B, Mertens A, Cho YM, Christiansen E, Hertz CL (2019). Efficacy and safety of oral semaglutide with flexible dose adjustment versus sitagliptin in type 2 diabetes (PIONEER 7): a multicentre, open-label, randomised, phase 3a trial. The lancet Diabetes & endocrinology.

[3] Heyman SN, Raz I, Dwyer JP, Weinberg Sibony R, Lewis JB, Abassi Z (2022). Diabetic Proteinuria Revisited: Updated Physiologic Perspectives. Cells.

[4] Liu D, Lv LL (2019). New Understanding on the Role of Proteinuria in Progression of Chronic Kidney Disease. Advances in experimental medicine and biology.

[5] Fioretto P, Pontremoli R (2022). Expanding the therapy options for diabetic kidney disease. Nature reviews Nephrology.

[6] Zhou Y, Xiong M, Fang L, Jiang L, Wen P, Dai C (2013). miR-21-containing microvesicles from injured tubular epithelial cells promote tubular phenotype transition by targeting PTEN protein. The American journal of pathology.

[7] Liu X, Liu Z, Liu Y, Ren Q, Jia N, Zhou L (2023). Kidney tubular epithelial cells control interstitial fibroblast fate by releasing TNFAIP8-encapsulated exosomes. Cell death & disease.

[8] Tsai YC, Hung WW, Chang WA, Wu PH, Wu LY, Lee SC (2021). Autocrine Exosomal Fibulin-1 as a Target of MiR-1269b Induces Epithelial-Mesenchymal Transition in Proximal Tubule in Diabetic Nephropathy. Frontiers in cell and developmental biology.

[9] Kalluri R, LeBleu VS (2020). The biology, function, and biomedical applications of exosomes. Science (New York, NY).

[10] Lv LL, Feng Y, Tang TT, Liu BC (2019). New insight into the role of extracellular vesicles in kidney disease. Journal of cellular and molecular medicine.

[11] Kosanović M, Llorente A, Glamočlija S, Valdivielso JM, Bozic M (2021). Extracellular Vesicles and Renal Fibrosis: An Odyssey toward a New Therapeutic Approach. International journal of molecular sciences.

[12] Jia Y, Zheng Z, Xue M, Zhang S, Hu F, Li Y (2019). Extracellular Vesicles from Albumin-Induced Tubular Epithelial Cells Promote the M1 Macrophage Phenotype by Targeting Klotho. Molecular therapy: the journal of the American Society of Gene Therapy.

[13] Jia Y, Chen J, Zheng Z, Tao Y, Zhang S, Zou M (2022). Tubular epithelial cell-derived extracellular vesicles induce macrophage glycolysis by stabilizing HIF-1α in diabetic kidney disease. Molecular medicine (Cambridge, Mass).

[14] Feng Y, Zhong X, Tang TT, Wang C, Wang LT, Li ZL (2020). Rab27a dependent exosome releasing participated in albumin handling as a coordinated approach to lysosome in kidney disease. Cell death & disease.

[15] van Niel G, D'Angelo G, Raposo G (2018). Shedding light on the cell biology of extracellular vesicles. Nature reviews Molecular cell biology.

[16] Sun W, Wang Y, Zheng Y, Quan N (2020). The Emerging Role of Sestrin2 in Cell Metabolism, and Cardiovascular and Age-Related Diseases. Aging and disease.

[17] Jia Y, Zheng Z, Yang Y, Zou M, Li J, Wang L (2019). MiR-4756 promotes albumin-induced renal tubular epithelial cell epithelial-to-mesenchymal transition and endoplasmic reticulum stress via targeting Sestrin2. Journal of cellular physiology.

[18] Liu Y, Li M, Du X, Huang Z, Quan N (2021). Sestrin 2, a potential star of antioxidant stress in cardiovascular diseases. Free radical biology & medicine.

[19] Li Y, Zhang J, Zhou K, Xie L, Xiang G, Fang M (2021). Elevating sestrin2 attenuates endoplasmic reticulum stress and improves functional recovery through autophagy activation after spinal cord injury. Cell biology and toxicology.

[20] Bae J, Jang Y, Kim H, Mahato K, Schaecher C, Kim IM (2019). Arsenite exposure suppresses adipogenesis, mitochondrial biogenesis and thermogenesis via autophagy inhibition in brown adipose tissue. Scientific reports.

[21] Bae SH, Sung SH, Oh SY, Lim JM, Lee SK, Park YN (2013). Sestrins activate Nrf2 by promoting p62-dependent autophagic degradation of Keap1 and prevent oxidative liver damage. Cell metabolism.

[22] Zhang XW, Zhou JC, Peng D, Hua F, Li K, Yu JJ (2020). Disrupting the TRIB3-SQSTM1 interaction reduces liver fibrosis by restoring autophagy and suppressing exosome-mediated HSC activation. Autophagy.

[23] Bai S, Hou W, Yao Y, Meng J, Wei Y, Hu F (2022). Exocyst controls exosome biogenesis via Rab11a. Molecular therapy Nucleic acids.

[24] Liu X, Tan S, Liu H, Jiang J, Wang X, Li L (2023). Hepatocyte-derived MASP1-enriched small extracellular vesicles activate HSCs to promote liver fibrosis. Hepatology (Baltimore, Md).

[25] Ma Q, Yu J, Liu L, Ma X, Zhang J, Zhang J (2023). TRAF6 triggers Mycobacterium-infected host autophagy through Rab7 ubiquitination. Cell death discovery.

[26] Ghilarducci K, Cabana VC, Harake A, Cappadocia L, Lussier MP (2022). Membrane Targeting and GTPase Activity of Rab7 Are Required for Its Ubiquitination by RNF167. International journal of molecular sciences.

[27] Wu M, Zhang M, Zhang Y, Li Z, Li X, Liu Z (2021). Relationship between lysosomal dyshomeostasis and progression of diabetic kidney disease. Cell death & disease.

[28] Yan S (2022). Role of TFEB in Autophagy and the Pathogenesis of Liver Diseases. Biomolecules.

[29] Buratta S, Tancini B, Sagini K, Delo F, Chiaradia E, Urbanelli L (2020). Lysosomal Exocytosis, Exosome Release and Secretory Autophagy: The Autophagic- and Endo-Lysosomal Systems Go Extracellular. International journal of molecular sciences.

[30] Nakamura J, Yamamoto T, Takabatake Y, Namba-Hamano T, Minami S, Takahashi A (2023). TFEB-mediated lysosomal exocytosis alleviates high-fat diet-induced lipotoxicity in the kidney. JCI insight.

[31] Liu BC, Tang TT, Lv LL, Lan HY (2018). Renal tubule injury: a driving force toward chronic kidney disease. Kidney international.

[32] Shen S, Ji C, Wei K (2022). Cellular Senescence and Regulated Cell Death of Tubular Epithelial Cells in Diabetic Kidney Disease. Frontiers in endocrinology.

[33] Bian Y, Shi C, Song S, Mu L, Wu M, Qiu D (2022). Sestrin2 attenuates renal damage by regulating Hippo pathway in diabetic nephropathy. Cell and tissue research.

[34] Molitoris BA, Sandoval RM, Yadav SPS, Wagner MC (2022). Albumin uptake and processing by the proximal tubule: physiological, pathological, and therapeutic implications. Physiological reviews.

[35] Liu D, Wen Y, Tang TT, Lv LL, Tang RN, Liu H (2015). Megalin/Cubulin-Lysosome-mediated Albumin Reabsorption Is Involved in the Tubular Cell Activation of NLRP3 Inflammasome and Tubulointerstitial Inflammation. The Journal of biological chemistry.

[36] Ishihara M, Urushido M, Hamada K, Matsumoto T, Shimamura Y, Ogata K (2013). Sestrin-2 and BNIP3 regulate autophagy and mitophagy in renal tubular cells in acute kidney injury. American journal of physiology Renal physiology.

[37] Yong CQY, Tang BL (2019). Another longin SNARE for autophagosome-lysosome fusion-how does Ykt6 work?. Autophagy.

[38] Tian X, Teng J, Chen J (2021). New insights regarding SNARE proteins in autophagosome-lysosome fusion. Autophagy.

[39] Bonam SR, Wang F, Muller S (2019). Lysosomes as a therapeutic target. Nature reviews Drug discovery.

[40] Quan N, Li X, Zhang J, Han Y, Sun W, Ren D (2020). Substrate metabolism regulated by Sestrin2-mTORC1 alleviates pressure overload-induced cardiac hypertrophy in aged heart. Redox biology.

