# Supplementary material for: SESN2 inhibits tubular exosome secretion and diabetic kidney disease progression by restoring the autophagy‒lysosome pathway
Source: Int J Biol Sci. 2025 Jun 20;21(9):4215–30. doi: 10.7150/ijbs.109799 (PMC12223778; doi:10.7150/ijbs.109799)
Supplement: Supplementary file 1 — Supplementary figures and tables. [file ijbsv21p4215s1.pdf]

Supplementary Figure1

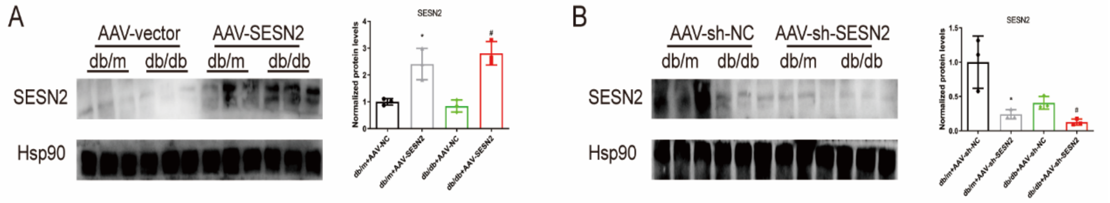

Sup 1: Efficacy of SESN2 overexpression or knockdown in mice. (A) Db/m and db/db mice were injected with AAV-vector or AAV-SESN2 via the tail vein, and protein expression levels of SESN2 in the renal cortex were measured (n=3). **p* < 0.05 vs. the db/m+AAV-vector group, #*p* < 0.05 vs. the db/db+AAV-vector group. (B) Db/m and db/db mice were injected with AAV-sh-NC or AAV-sh-SESN2 via the tail vein, and protein expression levels of SESN2 in the renal cortex were measured (n=3). **p* < 0.05 vs. the db/m+AAV-sh-NC group, #*p* < 0.05 vs. the db/db+AAV-sh-NC group.

Supplementary Figure2

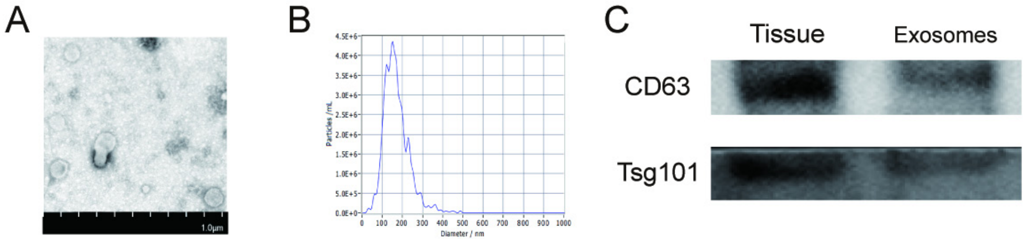

Sup 2: Characteristics of exosomes isolated from mice. (A) Representative TEM images of exosomes isolated from mice. Scale bars: 1.0 μm. (B) NTA analysis of the diameters of exosomes isolated from mice. (C) Detection of exosome markers in both tissue and exosomes.

Supplementary Figure3

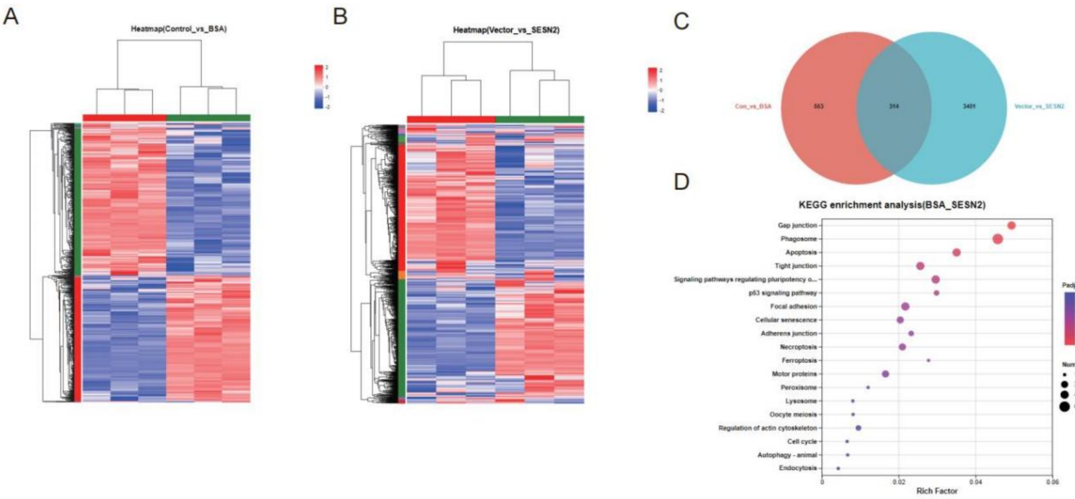

Sup 3:RNA-seq analysis of HK-2 cells treated with BSA or transfected with SESN2 (A) Heatmap of the differentially expressed genes in Control or BSA treated HK-2 cells. (B) Heatmap of the differentially expressed genes in Vector or SESN2 stably overexpressing HK-2 cells. (C)Venn diagram analyses of differentially expressed genes between Control_vs_BSA and Vector_vs_SESN2 groups. (D)KEGG pathway analysis according to the overlap filtering analysis results.

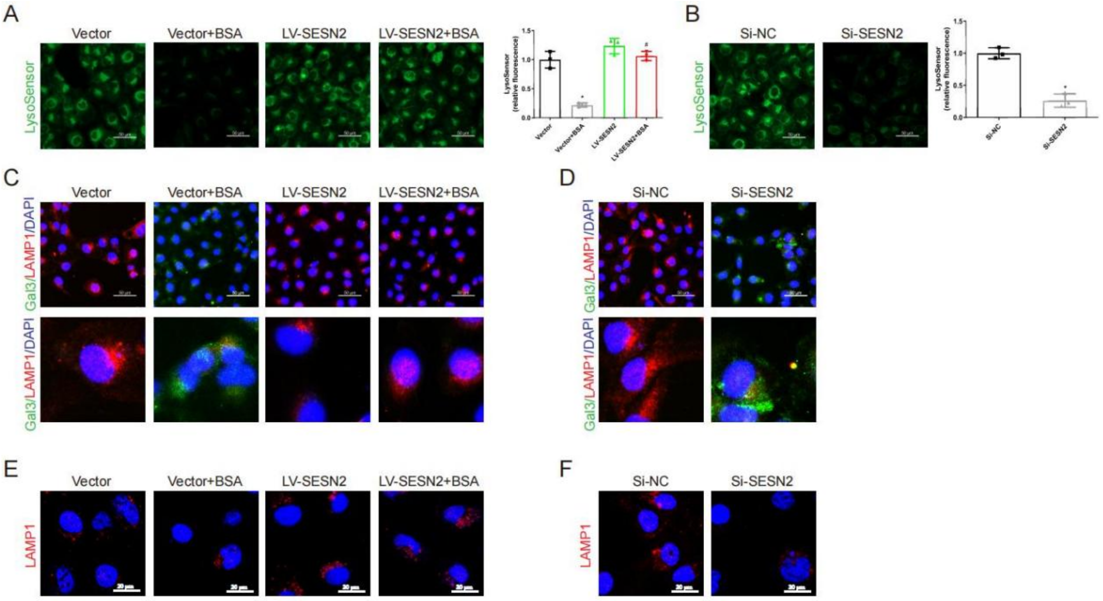

Sup 4: SESN2 ameliorates lysosomal stress and promotes lysosomal exocytosis. (A) Representative fluorescence microscopy images of Vector or SESN2 stably overexpressing HK-2 cells treated with or without BSA stained with LysoSensor. Scale bars: 50 μm. *$p < 0.05$ vs. the Vector group, #$p < 0.05$ vs. the Vector+BSA group. (B) Representative fluorescence microscopy images of Si-NC- or Si-SESN2-transfected HK-2 cells stained with LysoSensor. Scale bars: 50 μm. *$p < 0.05$ vs. the Si-NC group.(C) Representative immunostaining images of galectin-3 and LAMP1 in Vector or SESN2 stably overexpressing HK-2 cells treated with or without BSA. (D) Representative immunostaining images of galectin-3 and LAMP1 in Si-NC- or Si-SESN2-transfected HK-2 cells. Scale bars: 50 μm. (E) Representative immunostaining images of LAMP1 in Vector or SESN2 stably overexpressing HK-2 cells treated with or without BSA. (F) Representative immunostaining images of LAMP1 in Si-NC- or Si-SESN2-transfected HK-2 cells. Scale bars: 20 μm.

Supplementary Table 1: Results of molecular docking

Receptor	ligand	Binding energy	Interface area(Å^{2})	Hydrogen bonds (Rab7a:Sestrin2)
Rab7a	Sestrin2	-2.6 kcal/mol	1684.6	A:ARG 138 : C:TYR 349
				A:LYS 199 : C:PHE 447
				A:ASN 94 : C:SER 350
				A:ARG 113 : C:ASN 376
				A:ALA 200 ： C:ASN 247
				A:GLU 203 ： C:ASN 247
				A:GLU 203 ： C:ARG 445

Gene		5'-3'
hsa-TNF-α	Forward	AGCCTCTTCTCCTTCCTGAT
	Reverse	AAGATGATCTGACTGCCTGG
hsa-IL-6	Forward	ACTCACCTCTTCAGAACGAATTG
	Reverse	CCATCTTTGGAAGGTTCAGGTTG
hsa-FN	Forward	TAGCCCTGTCCAGGAGTTCA
	Reverse	CTGCAAGCCTTCAATAGTCA
hsa-Col-I	Forward	GATGGATTCCAGTTCGAGTATG
	Reverse	TGTTCTTGCAGTGGTAGGTGATG
hsa-α-SMA	Forward	TACTACTGCTGAGCGTGAGA
	Reverse	CATCAGGCAACTCGTAACTC
hsa-β-actin	Forward	TCGTGCGTGACATTAAGGAG
	Reverse	AGGAAGGAAGGCTGGAAGAG
mmu-TNF-α	Forward	CTTCTGTCTACTGAACTTCGGG
	Reverse	CACTTGGTGGTTTGCTACGAC
mmu-IL-6	Forward	TCCAGTTGCCTTCTTGGGAC
	Reverse	AGTCTCCTCTCCGGACTTGT
mmu-FN	Forward	CGAGGTGACAGAGACCACAA
	Reverse	CTGGAGTCAAGCCAGACACA
mmu- Col-1	Forward	ACATGTTCAGCTTTGTGGACC
	Reverse	TAGGCCATTGTGTATGCAGC
mmu-αSMA	Forward	TCCCTGGAGAAGAGCTACGAA
	Reverse	ATAGGTGGTTTCGTGGATGCC
mmu-β-actin	Forward	GGACTGTTACTGAGCTGCGTT
	Reverse	CGCCTTCACCGTTCCAGTT

Supplementary Table 2: Real Time PCR Primer Sets
